# Impacts of Telehealth Adoption on the Quality of Care for Individuals With Serious Mental Illness: Retrospective Observational Analysis of Veterans Affairs Administrative Data

**DOI:** 10.2196/56886

**Published:** 2024-07-09

**Authors:** Camilla Cummings, Pushpa Raja, Sonya Gabrielian, Neal Doran

**Affiliations:** 1 Veterans Affairs Greater Los Angeles Healthcare System, Los Angeles, CA, United States; 2 Department of Mental Health, Veterans Affairs Greater Los Angeles Healthcare System, Los Angeles, CA, United States; 3 David Geffen School of Medicine, University of California, Los Angeles, CA, United States; 4 Office of Mental Health, Veterans Affairs San Diego Healthcare System, San Diego, CA, United States

**Keywords:** telemedicine, quality of care, serious mental illness, telehealth, adoption, mental illness, patients, patient, veterans, veteran, psychotherapy, psychosocial, mental healthcare, suicide, rehabilitation, mental health care

## Abstract

**Background:**

Telehealth implementation can be challenging for persons with serious mental illness (SMI), which may impact their quality of care and health outcomes. The literature on telehealth’s impacts on SMI care outcomes is mixed, necessitating further investigation.

**Objective:**

We examined the impacts of facility-level telehealth adoption on quality of care metrics over time among patients with SMI.

**Methods:**

We analyzed Veterans Affairs (VA) administrative data across 138 facilities from January 2021 to December 2022. We performed longitudinal mixed-effects regressions to identify the relationships between the proportion of facility-level telehealth visits and SMI specialty care quality metrics: engagement with primary care; access and continuity of care across a range of mental health services including psychotherapy or psychosocial rehabilitation, SMI-specific intensive outpatient programs, and intensive case management; and continuity of mental health care after a high-risk event (eg, suicide attempt).

**Results:**

Facilities with a higher proportion of telehealth visits had reduced access and continuity of physical and mental health care for patients with SMI (*P*<.05). Higher telehealth adoption was associated with reduced primary care engagement (*z*=−4.04; *P*<.001), reduced access to and continuity in SMI-specific intensive case management (*z*=−4.49; *P*<.001; *z*=−3.15; *P*<.002), reductions in the continuity of care within psychotherapy and psychosocial rehabilitation (*z*=−3.74; *P*<.001), and continuity of care after a high-risk event (*z*=−2.46; *P*<.01). Telehealth uptake initially increased access to intensive outpatient but did not improve its continuity over time (*z*=−4.47; *P*<.001). Except for continuity within SMI-specific intensive case management (*z*=2.62; *P*<.009), continuity did not improve over time as telehealth became routinized.

**Conclusions:**

Although telehealth helped preserve health care access during the pandemic, telehealth may have tradeoffs with regard to quality of care for some individuals with SMI. These data suggest that engagement strategies used by SMI-specific intensive case management may have preserved quality and could benefit other settings. Strategies that enhance telehealth implementation—selected through a health equity lens—may improve quality of care among patients with SMI.

## Introduction

Telehealth accelerated during the COVID-19 pandemic and is likely to persist. Although telehealth is accepted by most psychiatric patients, individuals with primary or co-occurring serious mental illness (SMI; ie, psychotic spectrum disorders and bipolar disorders) show lower engagement [1,2] and are less likely to use video-based services [3,4] than other diagnostic groups. A systematic review found that individuals with (vs without) SMI use telehealth at lower rates, despite much higher in-person mental health care (MH) use among individuals with SMI [2]. A study of patients with SMI who use telehealth during the pandemic found that, although many patients with SMI maintained engagement, individuals with schizophrenia were the least likely to engage in telehealth [3]. Despite lower telehealth engagement among patients with SMI (defined variably in the literature, but often equated to the aforementioned diagnostic categories [5]), few have evaluated the impact of telehealth expansion on this population’s quality of care.

Literature on telehealth’s impacts on care outcomes for individuals with SMI is mixed. Prepandemic, county-level research linked telehealth uptake to modest increases in the proportion of patients with SMI receiving minimum levels of MH. Though telehealth resulted in a greater likelihood of posthospitalization follow-up, no changes in medication adherence and slight increases in acute care use were seen [6]. During the first 6 months of the pandemic, rapid telehealth growth was associated with lower MH use among individuals with SMI [7]. A recent study of specialty mental health practices with higher telehealth use found that Medicare patients with SMI had more visits per year, but no differences in medication refills, postpsychiatric hospitalization follow-up, or all-cause mortality [8].

The Department of Veterans Affairs (VA), which serves more patients with SMI than any other US health care system, is an opportune setting to examine telehealth’s impact on quality of care. VA maintains a comprehensive performance measurement system for outcome monitoring and quality improvement at national and facility levels [9]. This report analyzes national VA administrative data to characterize the impacts of facility-level telehealth adoption on SMI performance metrics, and interaction effects on quality metrics over time as telehealth was routinized.

## Methods

### Overview

National VA administrative data (from Corporate Data Warehouse) were used to extract all outpatient visits (across specialties) with SMI-specific *International Statistical Classification of Diseases, Tenth Revision* (*ICD-10*) codes as primary or secondary diagnostic codes. In total, 6 of 8 SMI-specific VA performance measures were examined; 2 measures had an expanded definition of SMI, encompassing visits with *ICD-10* codes for severe depression and posttraumatic stress disorder. Visits were stratified by modality (video telehealth vs face to face) and quarter from January 2021 to December 2022.

SMI-related performance metrics were collated across 138 VA facilities. Eight measures assessing access to care and care continuity for individuals with SMI were examined: (1) engagement with primary care (PC; ie, assigned to PC provider and ≥1 PC visit within the last year); (2) access to psychotherapy or psychosocial services (ie, proportion of patients with SMI who visited ≥1 psychotherapy or psychosocial rehabilitation within the last year); (3) continuity (ie, 5 psychotherapy or psychosocial rehabilitation visits within 10 weeks); access to care within 2 SMI specialty programs: (4) intensive outpatient programs (IOPs; ie, ≥3 visits in the past year); (5) intensive case management (ICM; VA’s brand of Assertive Community Treatment; ie, ≥5 visits in the past year); continuity in these SMI specialty programs, with (6) ≥3 IOP visits over 90 days and (7) ≥12 ICM visits over 90 days; and (8) continuity of MH after a high-risk event like psychiatric hospitalization or suicide attempt (ie, ≥1 visit per 6 months over the past year).

Stata (version 15.0; StataCorp LLC) was used to perform longitudinal mixed-effects regressions evaluating whether facility unstandardized scores changed in relation to total volume of SMI telehealth visits. We controlled for facility size approximated by facility-level volume of SMI visits and time (as telehealth was routinized after the initial pandemic emergency). Separate models were fit for each measure. Models initially included linear and quadratic time to assess whether scores on quality measures varied across the 2-year period. Initial models included all 2-way interactions between linear and quadratic time and total SMI visits and telehealth SMI visits to evaluate whether associations between SMI visit volume and quality measures changed over time. Nonsignificant interactions were not retained.

### Ethical Considerations

Procedures were approved by the VA San Diego Institutional Review Board (V201253). As an operations/quality improvement project, these data were collected in routine clinical care. Informed consent was not required, and no compensation was provided.

## Results

No interactions were observed between time and SMI visit volume, and interactions were omitted. Table 1 displays our findings. In total, 7 of 8 measures (all but access to psychotherapy) were significantly associated (*P*<.05) with total SMI volume, telehealth SMI volume, or both.

Four measures were positively associated with facility total SMI volume but negatively associated with facility telehealth volume, including continuity of psychotherapy or psychosocial care (*z*=−3.74), PC engagement (*z*=−4.04), and ICM program access and continuity (*z*=−4.49; *z*=−3.15). Access to IOPs was negatively associated with total SMI visit volume but positively with telehealth volume. Total SMI visit volume was negatively associated with continuity of MH after high-risk events (*z*=−2.46) and positively associated with continuity of IOP (*z*=2.24); neither measure was associated with telehealth visit volume.

In total, 6 of 8 measures changed over time. Access to psychotherapy, PC engagement, and continuity of MH after high-risk events were positively associated with linear time (*z*=4.39 or 2.54 or 8.18) but negatively with quadratic time (*z*=−3.78 or −2.34 or −6.37); these 3 measures demonstrated an inverted U shape over time, with initial improvement followed by a decline back toward baseline. Continuity of psychotherapy (*z*=−2.99) and IOP (*z*=−4.47) declined significantly and linearly over time, whereas continuity of ICM (*z*=2.62) exhibited a linear increase over time.

**Table 1.** Summary of models testing associations between SMI^a^ visits by modality and unstandardized scores on SMI-related quality measures across 138 Veterans Affairs facilities nationwide. *z* scores greater than 0 indicate a positive association between the quality measure and the time- or SMI visit volume–based predictor; *z* scores less than 0 reflect a negative association.

Quality measure	Definition	Linear time		Quadratic time		Total SMI visits^b^		Telehealth SMI visits	
		*z* score	*P* value	*z* score	*P* value	*z* score	*P* value	*z* score	*P* value
Access to psychotherapy	≥1 visit within past year	4.39	<.001	−3.78	<.001	−0.10	.92	0.46	.64
Continuity of psychotherapy	≥5 visits within 10 weeks past year	−2.99	.003	—^c^	—	6.15	<.001	−3.74	<.001
Access to primary care	Assigned primary care team +≥1 primary care visit past 12 months	2.54	.01	−2.34	.02	2.12	.03	−4.04	<.001
Continuity of mental health care after high risk event	≥1 visit per 6 months in past year	8.18	<.001	−6.37	<.001	−2.46	.01	0.46	.65
Access to ICM^d^	≥5 visits within past year	−0.30	.76	—	—	2.22	.03	−4.49	<.001
Continuity of ICM	≥12 visits within 90 days	2.62	.009	—	—	3.88	<.001	−3.15	.002
Access to IOP^e^	≥3 visits past year	−0.46	.65	—	—	−4.62	<.001	2.66	.008
Continuity of IOP^f^	≥3 visits within 90 days	−4.47	<.001	—	—	2.24	.02	−1.58	.11

a SMI: serious mental illness.

b SMI encompassing schizophrenia and other psychotic disorders and bipolar disorders.

c Not applicable.

d ICM: intensive case management.

e IOP: intensive outpatient program.

f Measures for access to and continuity of IOP include a broader definition of SMI including severe depressive disorders and posttraumatic stress disorder.

## Discussion

### Principal Findings

In VA facilities with higher telehealth adoption, patients with SMI had diminished engagement with PC, lower access and care continuity in ICM programs, and reduced psychotherapy or psychosocial continuity. Although telehealth uptake initially increased access to IOP care for patients with SMI, IOP continuity was not improved. Larger sites, measured by total SMI volume, performed better in PC engagement, access and continuity for ICM, and continuity of psychotherapy, adjusted for telehealth use.

Higher telehealth adoption at the facility-level may have reduced access to physical and MH for patients with SMI. Although telehealth helped preserve health care access during COVID-19, these findings suggest potential tradeoffs in quality metrics for individuals with SMI, particularly in PC engagement and continuity of care in psychotherapy or psychosocial rehabilitation settings and ICM programs. Though others report that practice-level telehealth use was associated with more visits per year for individuals with SMI [8], a subset of patients with SMI may require more robust supports (eg, from peers or case managers) to use telehealth without reducing access. Training and technical support may be necessary to overcome barriers such as hardware and software needs, broadband access, technological illiteracy, and condition-related aversions to technology mediated communication (eg, delusions) [10,11].

Access to primary and MH as well as continuity of MH after a high-risk event initially improved but returned to baseline over time. Continuity of psychotherapy and IOP declined over time. These findings suggest quality metrics did not improve as service providers and patients habituated to telehealth. Perhaps flexibility with modality and platform decreased as telehealth was routinized, or patients open to telehealth as a temporary measure were less open to it long term. The only metric to improve over time was continuity of SMI-specific ICM, suggesting more intensive strategies (eg, frequent in-person case management visits) may have facilitated successful engagement. Such strategies could be scaled to support telehealth implementation for SMI populations in other settings.

In addition to condition-specific barriers to care, individuals with SMI face social and economic inequities, including lower socioeconomic status, education, employment, and higher rates of homelessness, affecting their ability to engage in telehealth [12]. Access and continuity of care are crucial for this group because of their impact on health outcomes and mortality [13]. Our data suggest that reliance on telehealth may exacerbate disparities in health care access and quality, raising concerns about health equity for this vulnerable population.

These analyses are limited by a focus on VA, which differs from community systems in services and patient population. VA’s ICM and IOP programs have programmatic elements and performance metrics that may differ from other settings. These analyses used operations data intended for quality improvement; facility-level characteristics (eg, patient complexity) that could explain associations were not available. Future research could explore the impact of characteristics related to facility (eg, urbanicity or rurality), patients (eg, average age or facility), organization (eg, staffing), and provider attitudes regarding telehealth. Such investigations might assess the extent to which quality of care is impacted by telehealth enabling access to individuals previously disengaged from services altogether.

### Conclusions

These findings raise concerns about facility-level impacts of telehealth adoption on quality of care and service engagement for individuals with SMI. Though telehealth is effective for persons with SMI [14], it may contribute to quality gaps for some individuals with SMI, negatively impacting facility-level quality metrics. Strategic telehealth supports are likely needed to reduce inequities for individuals with SMI, such as telehealth literacy screening [15], telehealth skills training [16], or in-person support to enable telehealth access. Given existing health disparities among individuals with SMI [10,12], centering health equity in the identification of implementation supports is warranted to ensure equitable and effective service provision for this vulnerable population.

## Abbreviations

*ICD-10*: *International Statistical Classification of Diseases, Tenth Revision*
ICM: intensive case management
IOP: intensive outpatient program
MH: mental health care
PC: primary care
VA: Veterans Affairs
